# Affections of the salivary ducts in buffaloes

**Published:** 2014-06-22

**Authors:** N.A. Misk, T.N. Misk, M.A. Semieka, A.F. Ahmed

**Affiliations:** 1 *Department of Surgery, Radiology and Anesthesiology, Faculty of Veterinary Medicine, Assiut University, Assiut, Egypt*; 2 *Department of Surgery, Radiology and Anesthesiology, Faculty of Veterinary Medicine, Assiut University, Assiut, Egypt*

**Keywords:** Buffaloes, Ectasia, Fistula, Salivary duct, Sialocele

## Abstract

The aim of the present study was to determine different affections of the salivary ducts in buffaloes with special reference to diagnosis and treatment. The study was carried out on 39 buffaloes suffering from different affections of the salivary ducts. The recorded affections of the salivary ducts in buffaloes include; ectasia of the parotid duct (21 cases), parotid duct fistula (15 cases) and sialocele (3 cases). Each case was subjected to full study including case history, clinical examination, diagnosis, and treatment whenever possible. Exploratory puncture and radiography were used for confirmation of diagnosis. Intraoral marsupialization was performed for treatment of parotid duct ectasia. Salivary fistula was corrected by one of two successful techniques; the first by reconstruction of the parotid duct and the second by ligation of the parotid duct just caudal to the fistula opening. Sialoceles were corrected by removal of the mandibular salivary gland of the affected side.

## Introduction

The main salivary glands in buffaloes consist of three pairs namely; parotid, mandibular and sublingual glands.

The parotid duct emerges from the distal extremity of the gland and courses forward along the ventral border of the masseter muscle, then turns upward and gains the rostral border of the muscle. After a short course, the duct opens into the buccal vestibule at the parotid papilla (Barnwal and Sinha, 1982; Ahmed, 1988; Misk *et al.*, 1988).

The mandibular duct originates from the rostral border of the gland and continues rostrally between the digastric muscle and pterygoideus medialis muscle and then appears into the sublingual fold until it opens at the sublingual caruncle.

The sublingual duct originates from the rostral extremity of the gland and courses deeply with the mandibular duct until it opens alone or into a common duct with the mandibular duct at the sublingual caruncle (Getty, 1975; Dyce *et al.*, 1987; Tyagi and Singh, 1996).

The common affections of the salivary ducts in buffaloes were recorded in the available literatures. They include; ectasia of the parotid duct, salivary fistula, sialolith and sialoceles (Misk and Nigam, 1984; Misk *et al.*, 1991; Semieka, 2002; Sagar *et al.*, 2010).

The aim of the present study was to identify different affections of the salivary ducts in buffaloes with special reference to diagnosis and possible treatment.

## Materials and Methods

The present study was carried out on 39 buffaloes suffering from different affections of the salivary ducts. Cases were collected from the results of a project at the Faculty of Veterinary Medicine, Assiut University, Egypt (Misk, 2012). Three affections of the salivary ducts were recorded in the present study namely; ectasia of the parotid duct, parotid duct fistula and mandibular sialocele.

Ectasia of the parotid duct was diagnosed in 21 animals aged 4 months to 8 years. 15 animals were females and 6 were males. Diagnosis was established depending on the case history and clinical symptoms. Exploratory puncture and radiographic examination were used for confirmation of diagnosis. Marsupialization was the treatment of choice and was performed under the effect of the tranquilizer xylazine HCl 2% with a dose rate of 0.05 mg/kg intramuscularly.

Parotid duct fistula was diagnosed in 15 female buffaloes aged 2 to 8 years. Diagnosis was established depending on the presence of an opening along the course of the parotid duct discharging saliva. The condition was corrected by two techniques. The first one was applied in 6 animals and was performed by the application of a segment of polyethylene tube at the site of fistula for reconstruction of the patency of the salivary duct (Misk *et al.*, 1991). The second technique was applied in 9 animals and was performed by ligation of the salivary duct just caudal to the fistula opening under the effect of xylazine HCl 2% at a dose rate of 0.05 mg/kg intramuscularly (Greene and Thurmon, 1988) with subcutaneous local infiltration analgesia using 10 ml of lidocaine HCl 2% at the site of operation.

Sialocele was diagnosed in 3 adult female buffaloes. Diagnosis was established depending on the case history and clinical symptoms. Exploratory puncture was used for the confirmation of diagnosis. Aspiration of the contents from the swelling and extirpation of the mandibular salivary gland was performed in two animals. Follow up period of all cases was extended up to two months.

## Results

**Fig. 1:**
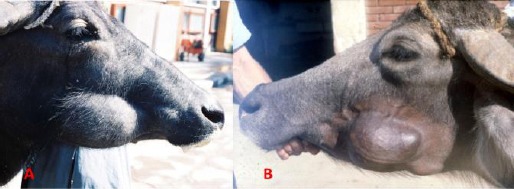
Parotid duct ectasia. **(A)** At the right cheek in a buffalo. **(B)** At the left cheek in a buffalo. Note the presence dilated tortuous parotid duct behind a fist size swelling.

Ectasia of the parotid duct was recorded in 21 animals (Figure 1).

The history of cases revealed occurrence of this condition in 13 calves up to one year old and in 8 adult buffaloes 1-8 years old. The parotid duct appeared as a dilated, variable size, soft tissue fluctuating swelling at the lateral aspect of the cheek. From the caudal aspect of the swelling, a dilated tortuous duct was seen directed to the ventral border of the gland behind the mandibular angle. Complete needle aspiration of the contents or hand pressure over the swelling led to its complete evacuation but refilling occurred within few minutes.

In all cases, marsupialization was performed through the mouth cavity after applying a suitable mouth gag. While an external hand pressure was applied on the swelling, a guarded curved lancet scalpel No. 22 was introduced into the mouth cavity and then a 2-3 cm vertical incision was made in the mucous membrane of the cheek through the dilated duct wall at the lowest part of the swelling.

**Fig. 2:**
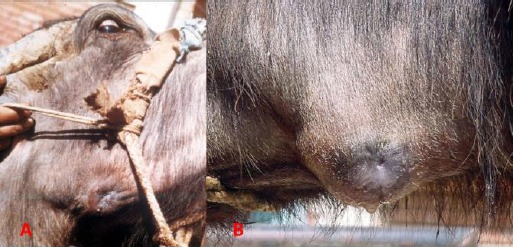
Parotid duct fistula in buffaloes. (**A** and **B**) Note in the presence of a tissue reaction around the fistula opening discharging saliva.

As the incision involved the wall of the duct, the contents of the swelling immediately entered the mouth cavity. The follow-up revealed complete recovery with gradual shrinkage of the cheek swelling. Recurrence of the condition was evident in one case after two weeks and the operation was repeated for the second time with successful results. Salivary parotid duct fistula was recorded in 15 adult buffaloes (Figure 2). Case history revealed the presence of long standing opening discharging watery secretion.

History of direct trauma or presence of abscess along the course of the duct was stated by animal’s owners. Callus tissue and severe dermatitis were noticed around the fistula opening.

**Fig. 3:**
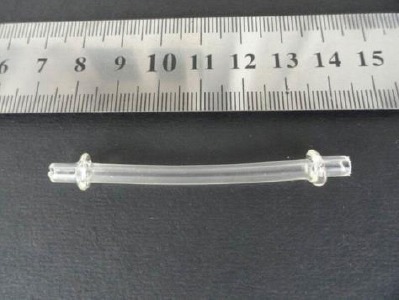
Polyethylene tube with a flange at both ends for partial reconstruction of the parotid duct

Two surgical techniques were used for treatment. The first technique was used in 6 cases by partial reconstruction of the parotid duct using a 10 cm segment of polyethylene tube with good results. The tube segment was prepared to have a flange at both ends (Figure 3) and simply introduced totally in a rostral direction through the fistula opening and was then pushed in the reverse direction until it slid past the opening in the duct, toward the gland. Two silk ligatures were applied blindly through the skin 2-3 cm rostral and caudal to the opening of the fistula. The skin edges at the site of fistula opening were freshened and trimmed and simple interrupted silk stitches were applied. Recovery was obtained in 5 out of 6 cases. The failed case was retreated using the second technique (ligation technique).

**Fig. 4:**
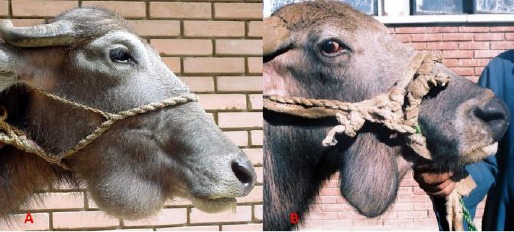
Sialocele in buffaloes. **(A)** Note the presence of a rounded swelling at the middle of mandibular space. **(B)** Note the presence of a vertical elongated swelling at the middle of mandibular space.

The second technique was the ligation of the parotid duct few cm caudal to the fistula opening. This technique was applied in 9 cases in addition to the failed case from group one. A metal probe was introduced through the fistula opening and directed caudally. After surgical preparation of the area caudal to the fistula, local infiltration of 10 ml lidocaine HCl 2% was performed along the course of the duct and a horizontal skin incision was made over the palpable probe. The parotid duct was bluntly dissected and two silk ligatures (# 2) were applied with 2 cm distance between them and tied after withdrawal of the probe. After that, the skin wound was closed using simple interrupted stitches (# 2). Also, the site of fistula opening was freshened and trimmed followed by simple interrupted silk stitches. Sialocele was recorded in 3 adult buffaloes (Figure 4).

A soft fluctuating painless swelling was detected at the mandibular space since a few months as per the case history. No case history was established explaining the beginning of the condition except a gradual appearance and enlargement of the swelling. Exploratory puncture revealed the presence of saliva and aspiration of the cystic swelling resulted in its collapse but refilling was detected within one hour or more.

Treatment was performed in only two cases, where unilateral removal of the mandibular salivary gland of the suspected affected side was performed in both cases. Recovery was uneventful in two cases and the third one was not operated at owner’s request.

## Discussion

Salivary ducts carry a huge amount of saliva from the salivary glands to the mouth cavity. Anatomically, the parotid salivary duct runs mostly subcutaneously along its course while mandibular and sublingual salivary ducts are embedded in tissues except at their terminal parts where they run submucosally inside the mouth cavity.

Any external trauma at the sides of the face may affect the parotid duct and lancing of an abscess at the head region may include the parotid duct leading to formation of salivary fistula. Chronic obstruction of the parotid duct may lead to its dilatation (ectasia) and formation of a cystic swelling at one side of the head. However, the presence of parotid duct ectasia in calves may have a congenital link (Misk *et al.*, 1991). Sialoceles occurs due to leakage of saliva from the injured mandibular or sublingual salivary ducts (Fubini and Ducharme, 2004). Intraoral trauma may cause the formation of sublingual or mandibular sialoceles (Misk, 2008). Diagnosis of salivary duct affections was performed depending on clinical presentation of all cases; however a degree of confusion may be present in the differential diagnosis between ectasia of the parotid duct and sialoceles (Table 1).

**Table 1:** Illustration of some points for differential diagnosis between parotid duct ectasia and sialocele

	Parotid duct ectasia	Sialocele
Location	The swelling at the cheek on one side of the face.	The swelling at the mandibular space.
Shape	Variable size swellings have a caudal tortuous tubular duct connected to the base of the gland.	Variable size swellings have no tubular connection with any gland.
Pressure over the swelling	Subsides completely leave a skin fold.	Remains as it is, without any changes.
The condition of the swelling after needle aspiration	Complete refilling within few minutes.	Complete refilling after one hour.

Treatment of ectasia of the parotid salivary duct was easily performed in all cases. Marsupialization was performed in standing position under xylazine sedation. A mouth gag was applied to facilitate the safe opening of the mouth cavity. Grasping of the tongue to the unaffected side protects it from accidental injury during operation. The surgical incision of the induced intraoral fistula must be performed at the lowest part of the swelling to facilitate drainage of the contents inside the mouth cavity. Continuous flow of saliva prevents healing of the intraoral incision and would lead to formation of a large permanent opening discharging saliva into the mouth cavity.

Complete reconstruction of the affected parotid duct in cases of parotid duct ectasia was performed in one buffalo with successful results. However, complete resection of the dilated duct and its replacement by a polyethylene tube is a tedious operation and should be performed only under general anesthesia (Misk *et al.*, 1991). Salivary fistula of the parotid duct was corrected successfully by reconstruction of the duct using a segment of polyethylene tube or by destruction of the gland through ligation of the duct just caudal to the fistula opening. Selection of either technique was based on the surgeon’s preference.

In addition, in cases of duct ligation, a weak-length distress results due to inflammation of the gland. From our point of view, reconstruction of the duct is preferable and should be performed first while ligation of the duct could be applied in unsuccessful cases (Jennings, 1984; Oehme and Prier, 1984).

The presence of sialoceles at the middle of mandibular space does not reveal the affected side. However sialography using contrast medium will help in differentiation of the healthy side from the affected side (Misk, 2008).

## Conclusion

The main affections of the salivary ducts in buffaloes are two; ectasia of the parotid duct and salivary fistula of the parotid duct while the third affection is mandibular sialoceles which occurs only sporadically. Fortunately, both common affections can be corrected easily, safely and in a short time without postoperative complications and with good prognosis.
